# Two-Year Follow-Up Study of the Relationship Between Brain Structure and Cognitive Control Function Across the Adult Lifespan

**DOI:** 10.3389/fnagi.2021.655050

**Published:** 2021-06-01

**Authors:** Shulan Hsieh, Meng-Heng Yang

**Affiliations:** ^1^Cognitive Electrophysiology Laboratory: Control, Aging, Sleep, and Emotion (CASE), Department of Psychology, National Cheng Kung University, Tainan, Taiwan; ^2^Institute of Allied Health Sciences, National Cheng Kung University, Tainan, Taiwan; ^3^Department of Public Health, National Cheng Kung University, Tainan, Taiwan

**Keywords:** cognitive control, aging, cross-sectional, longitudinal, gray matter

## Abstract

Age-related decline in cognitive control and general slowing are prominent phenomena in aging research. These declines in cognitive functions have been shown to also involve age-related decline in brain structure. However, most evidence in support of these associations is based on cross-sectional data. Therefore, the aim of this study is to contrast cross-sectional and longitudinal analyses to re-examine if the relationship between age-related brain structure and cognitive function are similar between the two approaches. One hundred and two participants completed two sessions with an average interval of 2 years. All participants were assessed by questionnaires, a series of cognitive tasks, and they all underwent neuroimaging acquisition. The main results of this study show that the majority of the conclusions regarding *age* effect in cognitive control function and processing speed in the literature can be replicated based on the cross-sectional data. Conversely, when we followed up individuals over an average interval of 2 years, then we found much fewer significant relationships between age-related change in gray matter structure of the cognitive control network and age-related change in cognitive control function. Furthermore, there was no “initial age” effect in the relationships between age-related changes in brain structure and cognitive function. This finding suggests that the “aging” relationship between brain structure and cognitive function over a short period of time are independent of “initial age” difference at time point 1. The result of this study warrants the importance of longitudinal research for aging studies to elucidate actual *aging* processes on cognitive control function.

## Introduction

Cognitive control is the ability to regulate one’s thoughts and actions on the basis of task goals. It refers to high-level executive functions, including selective attention involving inhibition of distractors, working memory, and task management (such as task switching and multitasking) (Miyake et al., 2000; Zanto and Gazzaley, 2017). Despite its vital role for humans to adapt quickly and accurately to changing environmental circumstances, its development and change paradoxically exhibit a “last in first out” phenomenon, in which it matures latest but declines earliest as compared with other cognitive functions (Craik and Bialystok, 2006). Meanwhile, it protracts development into early adulthood and a decline into older age which has also been shown to be associated with structural and functional changes in the prefrontal cortex (West, 1996; Raz et al., 1997). Most developmental and aging studies in the literature have investigated cognitive control differences across the lifespan (e.g., from early young adulthood to old age). They are interested in the question of whether different aspects of cognitive function are stable across adulthood and decline in older age or whether age-related declines in cognitive function begin soon after maturity in early adulthood. These previous studies have shown trajectories of functional decline in episodic memory, executive function, attention, and processing speed, but relatively preserved in their language and semantic knowledge (Light, 1991; Park, 2002; Park et al., 2002; Salthouse, 2004). However, the above evidence relies heavily on cross-sectional data, fewer on longitudinal data.

### Cross-Sectional (“Age”) vs. Longitudinal (“Aging”) Approach

Literature has highlighted the distinction between *age* effects and *aging* effects (e.g., Rugg, 2017). An age effect exists when a dependent variable *differs* among a sample of individuals with different mean ages (such as a younger group aged between 20 and 30 years, and an older group aged between 65 and 75 years)—this is known as a cross-sectional design. Conversely, an aging effect refers to an age-related difference in a dependent variable that can be attributed to the time-dependent *change* in the variable, i.e., to the “process” of aging—this is known as a longitudinal or follow-up design. Rugg (2017) has indicated a few inferring limitations regarding age-related cognitive aging based on the cross-sectional data. These limitations include that cross-sectional designs (1) confound aging, survivor, and birth cohort (e.g., Flynn effect) factors; (2) do not permit individual variation in cognitive performance or brain activity to be partitioned between age-invariant and age-related factors; (3) are difficult to infer about age-related changes in cognitive performance and their neural correlates; and (4) when to compare the fMRI blood-oxygen-level-dependency (BOLD), the results may be potentially confounded by differences in the hemodynamic response function. To consider these limitations, Hedden and Gabrieli (2004) directly compared cross-sectional and longitudinal data on various cognitive functions to see if there are different patterns. They observed linear age-related declines for speed, episodic memory, spatial ability, and reasoning in the cross-sectional data. On the other hand, they observed quite a different pattern in the longitudinal data, in which age-related changes from age 20 to 60 tended to be small or non-existent, with the speed of processing showing the largest change, whereas changes after the age of 60 had a slope that was roughly equivalent to that found in cross-sectional data. Their results implied the importance of longitudinal evidence to reveal actual *aging* effects in cognition.

### Brain Structure and Cognitive Control in Age/Aging

Cognitive control of aging has been shown to involve the decline of brain structure (Zanto and Gazzaley, 2017). Older adults have been shown to exhibit lower gray matter volumes (GMVs), particularly in the prefrontal cortex (PFC) and parietal cortex compared with younger adults (Gordon et al., 2008; Raz et al., 2010; Yang et al., 2019; Yao et al., 2020). Given that the PFC plays an important role in cognitive control function (Corbetta and Shulman, 2002; Cole et al., 2013; Yang et al., 2019; Yao et al., 2020), many theories of aging have hypothesized that the deterioration in PFC is the causal factor for age-related decline in cognitive control (Hasher and Zacks, 1988; Dempster, 1992; West, 1996; Reuter-Lorenz and Lustig, 2005; Hasher et al., 2007; Reuter-Lorenz and Cappell, 2008; Park and Reuter-Lorenz, 2009).

Likewise, the above evidence, showing GMVs associated with age-related differences in cognitive function, is mostly based on the cross-sectional design; relatively fewer studies (see, Oschwald et al., 2019 for a review) have provided direct evidence in showing brain structure in relation to age-related *changes*. Therefore, this study aims to fill the research gap by incorporating both cross-sectional and follow-up designs to re-examine the relationship between brain structure (e.g., GMV) and cognitive control function (e.g., shifting, inhibition, and working memory). In addition to cognitive control function, since general slowing is also a prominent phenomenon in aging research, we also incorporate processing speed in the analyses.

### The Present Study’s Aim and Rationale

This study aims to understand how differences vs. changes in GMV in the key areas of the cognitive control network (CCN; Breukelaar et al., 2017), such as PFC-associated structures, relate to differences vs. changes in cognitive control performance across the adult lifespan (e.g., 20–78 years). Please note, in this study, “aging” refers to the process of becoming older over time starting from any possible chronological age between 20 and 78 years and not necessarily restricted to the elderly age range (e.g., >65 years). Therefore, we recruited participants with an even age distribution between 20 and 80 years. We used the cross-sectional data at time point 1 (TP1) to first establish the relationship between (1) age and behavioral measures of cognitive control and processing speed, (2) age and brain structures that are related to the CCN, and then further examined if these cross-sectional relationships would be retained over time longitudinally. We also examined (3) the cross-sectional relationships between cognitive control function/processing speed and brain structures of the CNN, and also further examined (4) how the changes in brain structures of the CNN were associated with changes in cognitive control function/processing speed longitudinally.

Based on the previous studies, we predicted that a decrease in the GMV of the CNN would be associated with a decrease in the cognitive control ability and/or processing speed. Furthermore, if cross-sectional “age” differences could represent aging changes, we should then observe similar association patterns of GMV and cognitive performance between cross-sectional and longitudinal data, otherwise, their patterns would differ significantly.

## Materials and Methods

This study protocol was approved by the Human Research Ethics Committee of the National Cheng Kung University, Tainan, Taiwan, R.O.C. (Contract No. 104-004) to protect the participants’ rights according to the Declaration of Helsinki and the rule of research at the University. All participants signed an informed consent form before participating in the experiments.

### Participants

**FIGURE 1:**
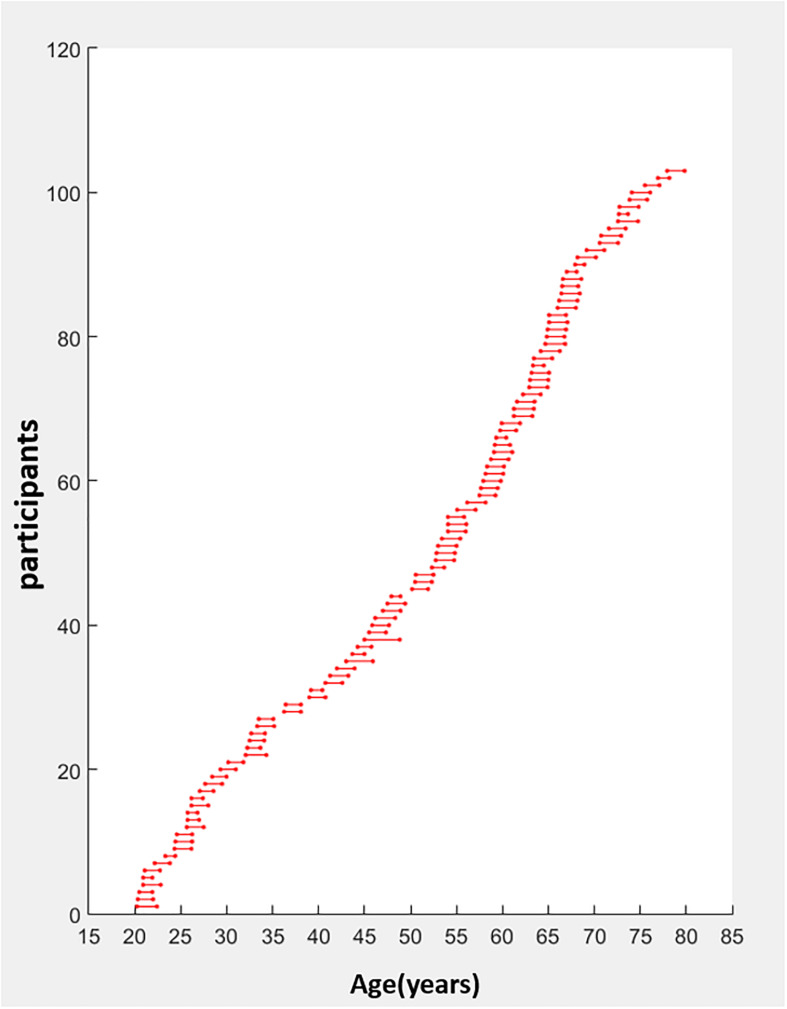
Age distribution of the longitudinal data (years). Participants aged 20–78 years old (*n* = 102) had a 3T MRI scan and completed cognitive tests at two time points, an average interval of 1.79 years apart (standard deviation = 0.37 year; range: 1–3.83 years). The age at each scan is indicated by a circle. The first scan is the leftmost with the repeated scan directly to the right and joined by a horizontal line.

We recruited 274 right-handed participants from southern Taiwan through advertisements on the Internet and bulletin boards. Only 107 participants completed two sessions with an average interval of 1.79 years apart (standard deviation = 0.37 year; range: 1–3.83 years). All participants were assessed by well-validated measures to evaluate their demographic information (e.g., level of education), cognitive (e.g., executive and motor functions), mental state (e.g., depression), quality of sleep, and multifaceted quality of life (e.g., positive feelings, social support, and financial status). They all underwent neuroimaging acquisition. Four participants were further excluded because of technical MRI problems or incomplete data. One additional participant was excluded because of the outliers in cognitive performance at TP1. The screening criteria for imaging quality control were based on head motion parameters and framewise displacement (FD). None of the remaining 102 participants’ max head motion exceeded 2.5 mm or mean FD exceeded 0.25. We also visually inspected all images after normalization and coregistration steps. This confirmed that there was no bad warping. The remaining 102 participants were all right handed and had no history of current psychological disorders or neurological disease. The mean age of the 102 participants (females = 63; males = 39) was 49.87 ± 16.99 years (20–29 years: *n* = 20; 30–49 years: *n* = 24; 50–64 years: *n* = 36; 65–78 years: *n* = 22). See Figure 1 and Table 1 for the participants’ age range distribution and demographic information.

**TABLE 1:** Demographic information and neuropsychological test results for the participants assessed at two time points.

	TP1	TP2	Paired *t* test (*p*)
*N*	102	102	N/A
Age	49.87 (±16.99)	51.66 (±17.08)	0.000***
MET	9.78 (±2.74)	9.84 (±2.70)	0.671
Sex (F%)	61.76%	61.76%	N/A
Education (years)	14.87 (±2.68)	14.87 (±2.68)	N/A
BDI-II	4.93 (±4.15)	4.88 (±5.04)	0.922
MoCA	27.50 (±1.86)	29.07 (±1.17)	0.000***
WHOQOL-BREF	57.89 (±6.72)	57.14 (±8.38)	0.146
PSQI	5.88 (±3.05)	6.18 (±3.48)	0.270

N, number of participants; TP1, time point 1; TP2, time point 2. Values reported as mean [±standard deviation (SD)]. Sex ratio calculated by numbers of female/male (F%); MET, metabolic equivalents test; BDI-II: Beck Depression Inventory-II; MoCA, Montreal Cognitive Assessment; WHOQOL-BREF, World Health Organization Quality of Life-Brief Version; PSQI, Pittsburgh Sleep Quality Index; N/A, not available. ***p < 0.0001.

### Cognitive Control Tasks

#### *N* (1 and 2)-Back Task

*N*-back task is commonly used to measure working memory updating function which was first introduced by Kirchner (1958). Participants were asked to complete 1-back and 2-back working memory tasks modified from the study of Jaeggi et al. (2008). In this task, the stimuli were presented within a 3^∗^3 grid in each trial. One of the grid squares was randomly assigned to be colored with blue. For the 1-back task, participants were asked to memorize the position of the blue grid square shown in the previous trials and to compare it with the position of the blue grid square in the current trial. For the 2-back task, participants were asked to memorize the position of the blue grid square in the previous two trials. If the blue grid square has appeared in the same location, participants pressed the “F” button using the left index finger. If the blue grid square has appeared in a different location, participants pressed the “J” button using the right index finger. The stimulus was presented for 500 ms and was followed by an interstimulus interval for 2,000 ms. Participants were told to respond before the next trial. The entire experiment contained one practice block with feedback and three formal blocks (21 trials per block). This task lasted for 15 to 20 min.

We calculated performance sensitivity (*d*′) as an index, which was based on the hit rate (*H*) and false-alarm (*F*) rate. The formula is *d*′ = *Z*(*H*) − *Z*(*F*) (*Z* denotes the *z* score of the normal distribution).

#### Stop-Signal Task

The stop-signal task is a test of inhibition of prepotent response which was introduced by Lappin and Eriksen (1966) and further developed by Gordon et al. (2008). In this study, we used a modified version of the paradigm by Verbruggen et al. (2008). The task stimulus (i.e., a symbol of “O” or “X”) was presented as a 2-cm (width) by 2-cm (height) white figure with a visual angle of 0.64° on a computer screen with a black background. Participants were instructed to perform a choice reaction time (RT) task (i.e., the go trials as the primary task), such as responding to the stimuli O or X by pressing the “z” or “/” button on a keyboard correspondingly with their left or right index finger. Occasionally (i.e., around a probability of 28.57%), the go stimulus was followed by an auditory stop signal (an approximate frequency of 1,000 Hz auditory “beep” sound appeared for 300 ms) after a stop-signal delay (SSD), which instructed participants to withhold their response (i.e., the stop trials: inhibiting the primary-task response given a stop signal). SSD was initially set at 150 and 350 ms and was adjusted continuously with the staircase tracking procedure, that is, the SSD would be increased by 50 ms if the previous stop trials was successfully inhibited, conversely, it would be decreased by 50 ms if the previous stop-trial was failed to be inhibited. Response registration continued during the stop-signal presentation. The interstimulus interval was approximately between 1,300 and 4,800 ms. Two practice blocks were first provided to participants to familiarize the task. After the two practice blocks, five experimental blocks commenced which contained randomly intermixing of 40 stop trials and 100 go trials (a probability of 28.57% for a stop signal) per block. The experimental blocks shared the same settings and response rules with the practice block. The completion time for the entire task was approximately 30 min.

The stop-signal reaction time (SSRT) was calculated by subtracting the median SSD from the median RT of the go trials and which was taken as an indicator of inhibition proficiency (the smaller, the better). The go trial’s RT in this task was taken as an index of general speed (see Table 2).

**TABLE 2:** Summary of the cognitive tasks’ performance indexes (*Z* scores) of cognitive function.

Task	Variable	Domain
TMT-A	Completion time	Speed
TMT-B	Completion time	Speed + shifting
(TMT-B-TMT-A)/TMT-A	Completion time ratio	Shifting
GPT_L/GPT_R	Completion time	Speed
Stop-signal task	SSRT	Inhibition
	goRT	Speed
Switching task	SWI cost (inform; non-inform)	Shifting
	MIX cost	Memory
1-Back task	*d*′	Memory
2-Back task	*d*′	Memory

**TMT-A, Trail Making Test—Form A; GPT_L, Grooved Pegboard Test, left hand; GPT_R, Grooved Pegboard Test, right hand; TMT-B, Trail Making Test-Form B; inform SWI cost, switch cost in informative cue condition; non-inform SWI cost, switch cost in non-informative cue condition; SSRT, Stop-signal reaction time; MIX cost, mixing cost derived from task-switching paradigm; 2-back d′, 2-back task’s sensitivity; 1-back d′, 1-back task’s sensitivity*.*

#### Task-Switching Paradigm

Task-switching abilities were measured by a modified paradigm from the study of Karayanidis et al. (2003). Each trial consisted of a cue and a target stimulus. Participants were instructed to respond to the target stimulus as quickly and as accurately as possible. There were two different cue conditions, i.e., informative and non-informative. The informative cueing condition contained two-color cues that informed of the forthcoming task: warm colors (red and orange) and cold colors (green and blue) which were assigned to a letter classification or a number classification task. For non-informative cue conditions, the cues were all colored in gray, which provided no information regarding the forthcoming task type. In this scenario, the subsequent target stimulus was presented in either a warm or a cold color (just like the informative cue colors) directly indicating which task to perform.

For both informative and non-informative cue conditions, there were two types of the target stimulus, incongruent and neutral. The incongruent target stimulus contained a pair of a Chinese letter and an Arabic number which were associated with different responses keys, whereas a neutral target stimulus contained a pair of one Chinese letter and a meaningless symbol (e.g., %#@$) or a pair of one Arabic number and a meaningless symbol. Chinese letters were selected from the Ten Celestial Stem system (i.e., Tiangan). The participants were asked to respond using their right and left index fingers mapped to the first-half/second-half for the Chinese-letter task or odd/even for the Arabic-number task. Cue-task mapping and hand-task mapping were counterbalanced across participants.

All participants practiced six blocks before the formal experiment. The formal experiment contained 12 blocks: (1) two single-task blocks of Arabic numbers for 70 trials per block; (2) two single-task blocks of Chinese letters for 70 trials per block; (3) four mixed informative task blocks for 70 trials per block; and (4) four mixed non-informative task blocks for 70 trials per block. The entire experiment lasted for about 35 ∼ 40 min.

We calculated the *informative switch cost* by subtracting the mean RT of the repeat trials from the mean RT of the switch trials in the mixed informative task blocks. We also calculated the *non-informative switch cost* by subtracting the mean RT of the repeat trials from the mean RT of the switch trials in the mixed non-informative task blocks. Switch cost is an index of shifting proficiency, which has been hypothesized to reflect either task-set reconfiguration (Rogers and Monsell, 1995), passive decay (e.g., task-set inertia proposed by Allport et al., 1994; or task-set priming proposed by Allport and Wylie, 1999), goal updating/retrieval from long-term memory (Mayr and Kliegl, 2003), or task-set inhibition process (Mayr and Keele, 2000). We considered both informative and non-informative switch costs because the former might involve more preparatory control processes (Cooper et al., 2015). Regardless of which theory is more appropriate, we used both informative and non-informative switch costs to reflect shifting proficiency (the smaller, the better).

In addition to these switch costs, we also calculated the *mixing cost* by subtracting the mean RT of the repeat trials in the single-task block from the mean RT of the repeat trials in the mixed informative task blocks. Mixing cost is attributed to increased demands on working memory, greater task ambiguity, and/or failure to fully disengage the alternative task-set (e.g., Mayr, 2001; Meiran and Gotler, 2001). Although researchers have argued that mixing cost might not necessarily reflect working memory but reflect monitoring the task cued process (see Prior and MacWhinney, 2010), in this study, we treated mixing cost as an indicator of working memory (the smaller, the better).

### Trail Making Test

In this study, we used the Chinese version of the Trail Making Test (TMT), which consisted of two forms (A and B) of task conditions. The reliability of the Chinese version of the TMT has been reported by Wang et al. (2018). Form A consisted of digits numbers of “1” ∼ “25” randomly shown on an A4 paper. Form B consisted of digit numbers of “1” ∼ “12” and Chinese zodiac letters (“rat” to “pig”) were shown. Participants drew a line connecting these items in a sequence of “1”–“2”–“3”–…“25” in form A and an alternating sequence of “1”–“rat”–“2”–“ox”–…“12”–“pig” in form B. The time to complete the form (TMT-A, TMT-B) was recorded as a performance index. TMT-A was considered a reflecting processing speed, whereas TMT-B as an index of switching proficiency plus processing speed. To evaluate shifting ability *per se*, we calculated the TMT ratio by subtracting the completion time of TMT-A from that of TMT-B and then further divided it by that of TMT-A.

### Grooved Pegboard Test

Participants were asked to insert cylindrical metal pegs into 25 holes of a pegboard as quickly as possible. We tested participants’ left-hand and right-hand performance separately. The test began with the self-identified dominant hand (i.e., the right hand in this study), followed by the non-dominant hand. Participants were asked to insert pegs in the standardized order (from left to right for all rows when using the right hand and from right to left for all rows when using the left hand) and to use only one hand at a time. The total time to complete the test was recorded as an index of processing speed for each hand, respectively.

The performance indexes (all transformed into *z* scores) collected from the above series of cognitive tasks are summarized in Table 2, in which TMT-A, GPT, go RT in a stop-signal task are considered indexes for processing speed, TMT-B, TMT ratio, and switch cost (informative and non-informative) are considered indexes of shifting, SSRT is an inhibition index, whereas mixing cost derived from task-switching paradigm, and 1-/2-back *d*′ are considered indexes of working memory.

### Neuroimaging Acquisition and Analysis

#### Image Acquisition

A GE MR750 3T scanner (GE Healthcare, Waukesha, WI, United States) installed in Mind Research and Imaging Center at National Cheng Kung University (NCKU) was used to acquire all brain imaging.

High-spatial-resolution T1-weighted images were acquired with fast spoiled gradient echo (fast-SPGR) (TR/TE: 7.6 ms/3.3 ms; flip angle: 12°; FOV: 22.4^∗^22.4 cm^2^; thickness: 1 mm; matrices: 224^∗^224). A total of 166 axial slices was acquired in a scan time of 218 s.

#### Gray Matter Volume Processing

GMVs were estimated by FreeSurfer 5.3^1^ with an automated surface-reconstruction scheme described in previous well-established studies. Regions of interest (ROIs) of the cognitive control network (CCN) were extracted using neuroanatomical labels in the Desikan–Killiany Atlas^2^ to map on a cortical surface model. GMVs in each ROI of FreeSurfer’s Atlas was extracted from output aparc.stats files. There is a total of 12 ROIs of the CCN including dorsal anterior cingulate cortex (dACC) which contains bilateral (L, left; R, right) rostral anterior cingulate gyrus (rosAntCG), dorsolateral prefrontal cortex (DLPC) which contains pars opercularis of the inferior frontal gyrus (infF-parOPC), rostral middle frontal gyrus (rosMidF), and dorsal parietal cortex (DPC) which contains inferior parietal cortex (infP), superior parietal cortex (supP), and precuneus.

#### Statistical Analyses

Gray matter volumes and cognitive performance (*z* scores) were plotted against age to visually inspect for outliers at two time points. To evaluate relationships between age, GMV, and cognitive-behavioral measures (including processing speed and cognitive control function), we first tested cross-sectional correlations between (1) age and cognitive performance, (2) age and GMV, and (3) GMV and cognitive performance. We used sex, education, and BDI-II as covariates in (1) ∼ (3) correlation analyses. Furthermore, in the 3rd analysis, we also added covariate of age at TP1 in the analysis.

We then used longitudinal data to calculate ΔGMV and Δcognitive performance by subtracting GMV (or score) at TP1 from TP2 and dividing by the exact number of years in between the two time points (Breukelaar et al., 2017). The time between scans was adjusted to 2 years as the average time between acquisitions was 1.79 years. Hereafter, we described the two time points’ interval as an average of 2 years. The formula is as follows:

$$\left({\mathrm{Measure}}_{\mathrm{TP2}}-{\mathrm{Measure}}_{\mathrm{TP1}}\right)\times 2/\left(\mathrm{TP2}-\mathrm{TP1}\right)=\Delta \,\mathrm{measure}$$

We tested the longitudinal correlation between (1) age (TP1) and Δcognitive performance, (2) age (TP1) and ΔGMV, and (3) ΔGMV and Δcognitive performance. We used sex, education, and BDI-II as covariates in (1) ∼ (3) correlation analyses. Furthermore, in the 3rd analysis, we also added covariate of age at TP1 in the analysis.

All data were transformed into *z* score before correlation analyses in this study. For all analyses, Bonferroni correction for multiple comparisons was applied to a non-adjusted significance level of *p* < 0.05. As we analyzed a total of 12 ROIs of the CCN, a value of *p* < 0.004 (0.05/12) was considered significant. However, in the Section “Results,” we also report trend relationships that did not survive correction for multiple comparisons (e.g., 0.004 < *p* < 0.05).

## Results

### Cross-Sectional Analysis at TP1

#### Age and Cognitive-Behavioral Measures

Table 3 shows correlations between age and cognitive performance (including processing speed and cognitive control function). Age at TP1 was found to be positively correlated with processing speed (reflected on TMT-A, GPT_L, GPT_R, and goRT), shifting (TMT-B), and inhibition (SSRT) (note: the higher values in RTs for these measures, the lower performance they indicated) and negatively correlated with working memory (reflected on the 2-back *d*′) (Bonferroni corrected, *p* < 0.004). The results suggest that age was associated with cognitive control and processing speed decline cross-sectionally.

**TABLE 3:** The correlation between age at TP1 and cognitive performance (including processing speed and cognitive control function) for the cross-sectional data (*n* = 102).

		Cross-sectional cognitive performance (TP1) correlation with
Cognitive function	Task	Age (TP1)
Speed	TMT-A	0.531*
	GPT_L	0.602*
	GPT_R	0.548*
	goRT	0.386*
Shifting	TMT-B	0.399*
	TMT-B-TMT-B/TMT-A ratio	−0.094
	infSWIcost	−0.239
	Non-infSWIcost	0.080
Inhibition	SSRT	0.365*
Memory	MIXcost	−0.061
	2-Back *d*′	−0.397*
	1-Back *d*′	−0.185

*p < 0.004. TMT-A, Trail Making Test-Form A; GPT_L, Grooved Pegboard Test, left hand; GPT_R, Grooved Pegboard Test, right hand; TMT-B, Trail Making Test-Form B; infSWIcost, switch cost in informative cue condition; non-infSWIcost, switch cost in non-informative cue condition; SSRT, stop-signal reaction time; MIXcost, mixing cost in task switching paradigm; 2-back d′, 2-back task’s sensitivity; 1-back d′, 1-back task’s sensitivity.

#### Age and GMV

The left-most column of Table 4 shows correlation results between age and GMV at TP1, partialling out sex, education, and BDI-II scores. Age was found to be negatively correlated with all ROIs of the CCN, such as dACC, DLPFC, and DPC (Bonferroni corrected, *p* < 0.004).

**TABLE 4:** Cross-sectional correlation table (*n* = 102).

	Cross-sectional GMV (TP1) correlation with					Cross-sectional GMV(TP1) correlation with			
	Age	Behavior (covariate: sex, edu, and BDI-II)				Behavior (covariate: age, sex, edu, and BDI-II)			
		Speed	Shifting	Inhibition	Memory	Speed	Shifting	Inhibition	Memory
**dACC**									
rosAntCG_L	−0.394*	ns	0.295 (SWI)*	−0.221 (SSRT)	0.235 (1-back)	ns	0.225 (SWI)	ns	ns
rosAntCG_R	−0.286*	−0.284 (GPT_R)*; −0.247 (GPT_L)	ns	−0.231 (SSRT)	0.202 (2-back)	ns	0.261(non-infSWI)	ns	ns
**DLPFC**									
infF-parOPC_L	−0.467*	−0.266 (TMT-A); −0.269 (GPT_R); −0.241 (GPT_L)	−0.207 (TMTB)	−0.204 (SSRT)	0.238 (1-back)	0.289 (goRT)*	ns	ns	ns
infF-parOPC_R	−0.545*	−0.351 (TMT-A)*; −0.287 (GPT_R)*; −0.323 (GPT_L)*	ns	−0.281 (SSRT)	0.226 (1-back); 0.212 (2-back)	ns	ns	ns	ns
rosMidF_L	−0.638*	−0.270 (TMT-A); −0.400 (GPT_R)*; −0.397 (GPT_L)*; −0.211 (goRT)	0.206 (SWI)	−0.205 (SSRT)	0.372 (2-back)*	ns	ns	ns	ns
rosMidF_R	−0.603*	−0.214 (TMT-A); −0.321 (GPT_R)*; −0.317 (GPT_L)*	0.319 (SWI)*	ns	0.284 (2-back)*	ns	0.208 (TMT-B); 0.225 (SWI)	ns	ns
**DPC**									
infP_L	−0.420*	−0.312 (GPT_R)*; −0.279 (GPT_L)	ns	ns	0.224 (2-back)	ns	ns	ns	ns
infP_R	−0.517*	−0.328(TMT-A)*; −0.482 (GPT_R)*; −0.436 (GPT_L)*	−0.212 (TMT-B)	ns	0.268 (2-back)	−0.277 (GPT_R)	ns	ns	ns
supP_L	−0.458*	−0.271 (GPT_R); −0.319 (GPT_L)*	ns	ns	ns	0.263 (goRT)	ns	ns	ns
supP_R	−0.488*	−0.262 (GPT_R); −0.307(GPT_L)*	0.272 (SWI)	−0.294 (SSRT)*	0.211 (2-back)	ns	ns	ns	ns
precuneus_L	−0.514*	−0.296 (GPT_R)*; −0.338 (GPT_L)*	0.210 (SWI)	ns	0.256 (2-back)	ns	ns	ns	ns
precuneus_R	−0.453*	−0.238 (TMT-A); −0.364 (GPT_R)*; −0.382 (GPT_L)*	−0.213 (TMT-B); 0.201 (SWI)	ns	0.237 (2-back)	0.221 (goRT)	ns	ns	ns

*p < 0.004 (Bonferroni corrected); GMV, gray matter volume; edu, education; L, left; R, right hemisphere; dACC, dorsal anterior cingulate cortex; rosAntCG, rostral anterior cingulate gyrus; DLPFC, dorsolateral prefrontal cortex; infF-parOPC, pars opercularis of the inferior frontal gyrus; rosMidF, rostral middle frontal gyrus; DPC, dorsal parietal cortex; infP, inferior parietal cortex (infP); supP, superior parietal cortex; TMT-A, Trail Making Test-Form A; GPT_L, Grooved Pegboard Test, left hand; GPT_R, Grooved Pegboard Test, right hand; TMT-B, Trail Making Test-Form B; SWI, switch cost in informative cue condition; non-infSWI, switch cost in non-informative cue condition; SSRT, stop-signal reaction time; 2-back, 2-back task’s sensitivity; 1-back, 1-back task’s sensitivity.

#### Correlation of GMV and Cognitive-Behavioral Measures

Table 4 shows significant correlations (both corrected and uncorrected *p* results) between GMV and cognitive performance at TP1, either (1) partialling out the sex, education, and BDI-II scores or (2) partialling out age, sex, education, and BDI-II scores.

The results show that when we only partialled out the sex, education, and BDI-II scores, there were many significant associations and trend associations between GMV and cognitive performance in many ROIs of the CCN (Table 4, middle panel). However, if we additionally partialled out age, then most of the correlations disappeared, only goRT remained significantly correlated with parOPC_L (Bonferroni corrected *p* < 0.004), and there were some trends of associations between rosAntCG with shifting and between rosMidF_R with shifting (Table 4, right panel).

### Longitudinal Analysis

#### Correlation of Change in Cognitive-Behavioral Measures With Age at TP1

The correlation between change in cognitive performance (Δcognitive) with age at TP1 shows that only GPT_L had a significant relationship with age (Bonferroni corrected *p* < 0.004) (Table 5).

**TABLE 5:** The correlation between age at TP1 and cognitive performance (including processing speed and cognitive control function) for the longitudinal data (*n* = 102).

		Longitudinal Δcognitive performance correlation with
Cognitive function	Task	Age (TP1)
Speed	TMT-A	0.013
	GPT_L	0.354*
	GPT_R	0.100
	goRT	0.009
Shifting	TMT-B	−0.147
	TMT-B-TMT-B/TMT-A ratio	−0.179
	infSWIcost	0.137
	Non-infSWIcost	0.178
Inhibition	SSRT	−0.127
Memory	MIXcost	0.036
	2-Back *d*′	−0.071
	1-Back *d*′	0.040

*p < 0.004. TMT-A, Trail Making Test-Form A; GPT_L, Grooved Pegboard Test, left hand; GPT_R, Grooved Pegboard Test, right hand; TMT-B, Trail Making Test-Form B; infSWIcost: switch cost in informative cue condition; non-infSWIcost, switch cost in non-informative cue condition; SSRT, stop-signal reaction time; MIXcost, mixing cost in task switching paradigm; 2-back d′, 2-back task’s sensitivity; 1-back d′, 1-back task’s sensitivity.

#### Correlation of Change in (Δ) GMV With Age at TP1

**FIGURE 2:**
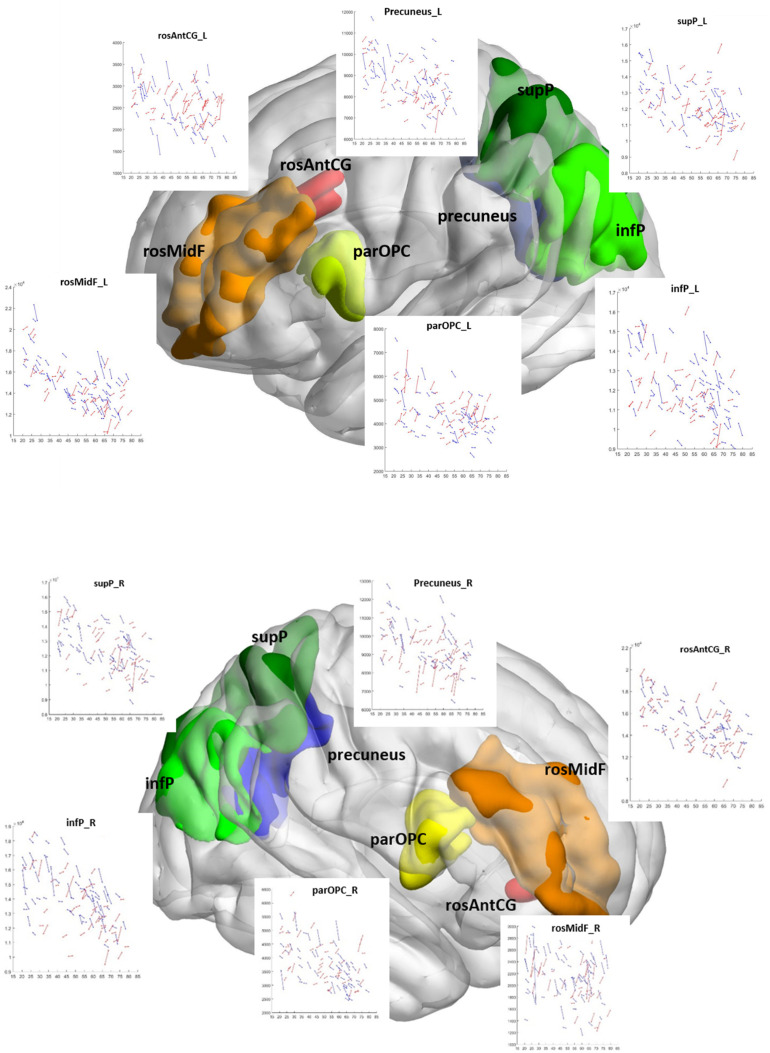
Change in gray matter volume with age at time point 1 in the cognitive control network. Longitudinal gray matter volume trajectories in all 12 ROIs of the cognitive control network are shown as scatter plots of gray matter volume (all *y*-axis) and age (all *x*-axis). Each scan is represented by a dot, and repeat scans are connected by lines. Coordinates of the CCN ROIs are plotted within a 3D brain: DLPFC: blue; dACC: yellow-red; DPC: green. L, left; R, right hemisphere; dACC, dorsal anterior cingulate cortex; rosAntCG, rostral anterior cingulate gyrus; DLPFC, dorsolateral prefrontal cortex; infF-parOPC, pars opercularis of the inferior frontal gyrus; rosMidF, rostral middle frontal gyrus; DPC, dorsal parietal cortex; infP, inferior parietal cortex (infP); supP, superior parietal cortex.

Figure 2 shows the change in GMVs with age at TP1 in each ROI of the CCN regardless of significance. In contrast to the cross-sectional results, only change in the rosAntCG_L had a significant relationship with age (Bonferroni corrected, *p* < 0.004; the left-most column in Table 6).

**TABLE 6:** Longitudinal correlation table (*n* = 102).

	Longitudinal ΔGMV correlation with					Longitudinal ΔGMV correlation with			
	Age (TP1)	BehaviorΔ (covariate: sex, edu, BDI-II)				BehaviorΔ (covariate: age, sex, edu, BDI-II)			
		Speed	Shifting	Inhibition	Memory	Speed	Shifting	Inhibition	Memory
**dACC**									
rosAntCG_L	0.290*	ns	Ns	ns	ns	ns	ns	ns	ns
rosAntCG_R	ns	ns	Ns	ns	0.221 (2-back)	ns	ns	ns	0.222 (2-back)
**DLPFC**									
infF-parOPC_L	ns	ns	Ns	0.201(SSRT)	ns	ns	ns	ns	ns
infF-parOPC_R	ns	ns	Ns	0.221 (SSRT)	0.285 (1-back)*	ns	ns	0.232 (SSRT)	0.283 (1-back)*
rosMidF_L	ns	ns	0.227 (noinfsw)	ns	ns	ns	0.221 (non-infswi)	ns	ns
rosMidF_R	ns	ns	0.224 (noinfswi)	ns	ns	ns	0.205 (non-infswi)	ns	ns
**DPC**									
infP_L	ns	ns	Ns	ns	ns	ns	ns	ns	ns
infP_R	ns	ns	Ns	ns	ns	ns	ns	ns	ns
supP_L	ns	ns	Ns	ns	0.272 (1-back)	ns	ns	ns	0.270 (1-back)
supP_R	ns	ns	Ns	ns	ns	ns	ns	ns	ns
precuneus_L	ns	ns	Ns	ns	ns	ns	ns	ns	ns
precuneus_R	ns	ns	Ns	ns	ns	ns	ns	ns	ns

*p < 0.004 (Bonferroni corrected); ΔGMV, changes in gray matter volume; edu, education; L, left; R, right hemisphere; dACC, dorsal anterior cingulate cortex; rosAntCG, rostral anterior cingulate gyrus; DLPFC, dorsolateral prefrontal cortex; infF-parOPC, pars opercularis of the inferior frontal gyrus; rosMidF, rostral middle frontal gyrus; DPC, dorsal parietal cortex; infP, inferior parietal cortex (infP); supP, superior parietal cortex; TMT-A, Trail Making Test-Form A; GPT_L, Grooved Pegboard Test, left hand; GPT_R, Grooved Pegboard Test, right hand; TMT-B, Trail Making Test-Form B; SWI, switch cost in informative cue condition; non-infSWI, switch cost in non-informative cue condition; SSRT, stop-signal reaction time; 2-back, 2-back task’s sensitivity; 1-back: 1-back task’s sensitivity.

#### Correlation of Change in Gray Matter Volume With Change in Cognitive-Behavioral Measures

**FIGURE 3:**
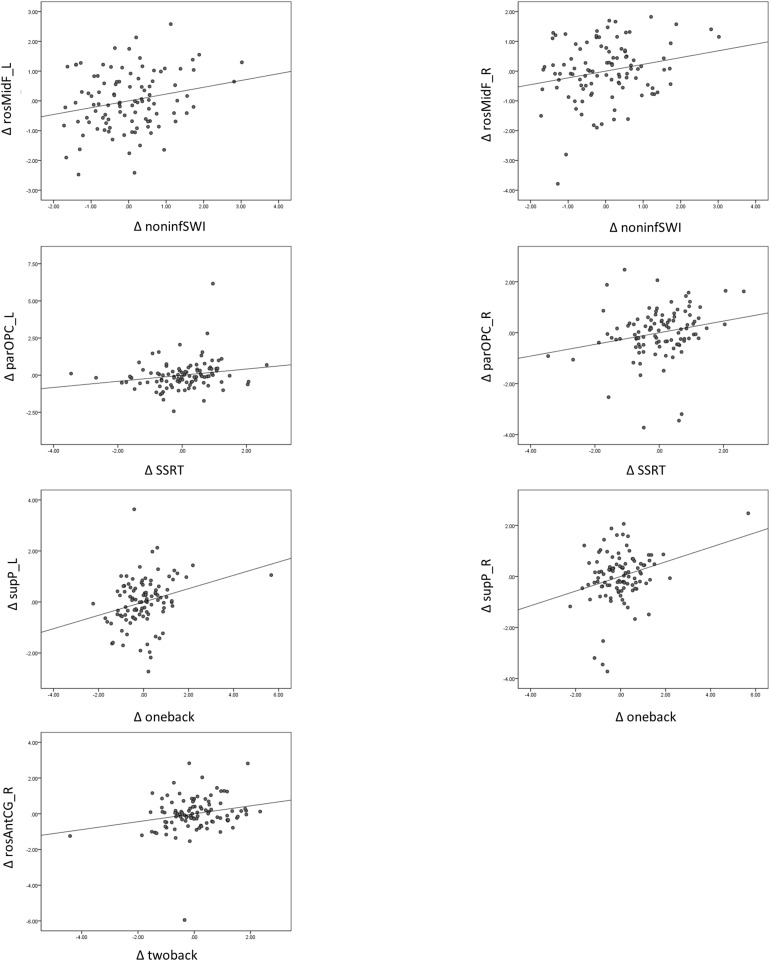
Relationship between change in gray matter volume (ΔGMV) and change in cognitive performance (Δ*Z*-score), controlling for sex, education, and BDI-II score, shows changes in GMV in the parOPC and rosAntCG_R and sup_L associated with 1-back *d*′, 2-back *d*′, and SSRT. L, left; R, right; rosMidF, rostral middle frontal; parOPC, pars opercularis; supP, superior parietal; rosAntCG, rostral anterior cingulate.

There was a significant relationship between change in (Δ) 1-back *d*′ and change in (Δ) parOPC_R (Bonferroni, *p* < 0.004) (Figure 3) when we only partialled out the sex, education, and BDI-II scores. We also found a few trend associations between ΔGMV and Δ*Z*-score of cognitive performance (e.g., rosMidF_L/R with non-inform switch cost; 2-back *d*′ with rosAntCG_R; 1-back *d*′ with supP_L, parOPC_R; SSRT with parOPC L/R) (Table 6, middle panel).

When we additionally controlled for age (TP1) in the analysis, the original significant and trend associations still remained the same (Table 6, right panel).

## Discussion

This study aims to contrast the results obtained from the cross-sectional vs. longitudinal data analyses regarding the relationships between age with cognitive control function/processing speed, between age with brain structure of the CCN, as well as between cognitive control function/processing speed with gray matter brain structure of the CCN.

In regard to the relationship between *age* and *cognitive control function/processing speed*, similar to those widely reported in the literature, we observed several significant associations between age differences at TP1 (i.e., cross-sectional approach) with several measures of cognitive control function (including shifting, inhibition, and working memory) and processing speed. However, these cross-sectional significant relationships *no longer* exist (except the processing speed indicated by GPT_L) when we examined the changes in these brain structures over time (i.e., longitudinal approach; see Table 3). These findings appear to be consistent with those reported by Hedden and Gabrieli (2004), in which they observed different patterns between cross-sectional and longitudinal data on various cognitive functions. Thus, the current results, in support of their arguments, advocate the importance of longitudinal evidence to reveal actual *aging* effects on cognition.

As for the relationship between *age* and *gray matter* brain structure of the CNN, the current results on the cross-sectional analyses showed likewise several significant associations between GMVs of dACC, DLPFC, and DPC with age at TP1 (see the left-most column in Table 4). This finding is consistent with most of the studies reported in the literature showing gray matter volume loss with age (see Oschwald et al., 2019 for a review). For example, Schippling et al. (2017) using two independent, cross-sectional single-scanner cohorts of healthy individuals have demonstrated that global and regional annual brain volume loss rates are consistent between the two cohorts and independent of the scanners^3^. However, on the contrary, the longitudinal data showed that only one brain region’s (i.e., rosAntCG_L) GMV changes over time were significantly associated with age at TP1 (see the left-most column in Table 6). The results again highlight the importance of longitudinal evidence to reveal actual *aging* effects in brain structure.

With regard to the relationships between cognitive control function/processing speed with gray matter brain structure of the CNN, the current cross-sectional data showed that several brain structures’ GMVs of the CNN (e.g., dACC, DLPFC, and DPC) differed significantly across age at TP1 (see columns 2–5 from the left in Table 4). For example, rostral anterior cingulate, pars opercularis of the inferior frontal gyrus, rostral middle frontal gyrus, inferior parietal cortex, superior parietal cortex, and precuneus were found to be associated with age-related differences in processing speed and cognitive control function, such as shifting (measured by the task-switching paradigm), inhibition (measured by the stop-signal task), and working memory (measured by 1- and 2-back tasks), respectively. These findings are consistent with literature based on cross-sectional data showing that brain structures within the cognitive control network are related to cognitive control function as well as processing speed (e.g., Fjell et al., 2009; Burzynska et al., 2012; Ruscheweyh et al., 2013; Ferreira et al., 2014; Arvanitakis et al., 2016; Bauer et al., 2018; Hu et al., 2018; Samrani et al., 2019).

However, of the main interest, when we further examined these relationships by mapping changes in gray matter brain structures of the CNN and changes in cognitive control performance/processing speed over time (i.e., the longitudinal approach), we then observed much fewer significant relationships between these two variables’ changes over time. For example, the results of these longitudinal analyses showed only one significantly positive association between GMV changes in DLPFC (e.g., right parOPC of the inferior frontal gyrus) and sensitivity changes in the 1-back task, and some trends of associations (i.e., uncorrected significance: 0.004 < *p* < 0.05) between changes in the left superior parietal associated with 1-back task’s sensitivity; rostral anterior cingulate associated with 2-back sensitivity; bilateral parOPC associated with inhibition; and bilateral rostral middle frontal associated with non-inform switch cost) (see columns 2–5 from left in Table 6). Although there is not much longitudinal evidence available in the literature, the current results are nevertheless consistent with those reported by the few available studies (e.g., Tisserand et al., 2004; Cardenas et al., 2011; Dicks et al., 2018; Vibha et al., 2018). Additionally, the current finding of a decrease in GMV in bilateral parOPC of the inferior frontal gyrus associated with a decrease in inhibition efficacy appears to be consistent with the literature showing a close relationship between the (right) inferior frontal gyrus and motor function based on the cross-sectional studies (e.g., Aron et al., 2004; Molnar-Szakacs et al., 2005; Hampshire et al., 2010; Falquez et al., 2014; Boen et al., 2020), as well as longitudinal findings (e.g., Breukelaar et al., 2017; Curley et al., 2018—yet with children samples).

More interestingly, these observed relationships between GMV changes and behavioral changes obtained from the follow-up analyses were further found to be independent of the initial age difference at TP1, since the correlation pattern remained the same when we further partialled out age (at TP1) factor (see the right panel in Table 6). This finding suggests that the aging relationship between brain structure and cognitive function over time is independent of the initial age difference at TP1. To summarize the major results of this study, we found that the majority of the conclusions regarding *age* effect in cognitive control function and processing speed in the literature are based on the cross-sectional data. Conversely, if we follow-up an individual over an average interval of adjusted 2 years, then we found there seemed to be no initial age (i.e., age at TP1) effect in the relationship between brain structure and cognitive function since across participants with different age at TP1 exhibited a similar degree of *aging* dynamic changes over a follow-up period.

The current findings add to the existing few research (e.g., Hedden and Gabrieli, 2004) concurrently examining cross-sectional and longitudinal data on cognitive functions across the adult lifespan. The data contributes toward bringing researchers’ attention to the discrepancies regarding cognitive control aging between cross-sectional and longitudinal approaches. While it has been widely believed that cognitive control function is sensitive to age and highly correlated with PFC-associated structures based on the cross-sectional data (e.g., Light, 1991; Park, 2002; Park et al., 2002; Salthouse, 2004), the current results, however, showed that cognitive control function did not exhibit accelerated decline for older people over a period of adjusted 2 years. This implies that cognitive control aging is more sophisticated than the original thought. It also highlights the possibilities of cohort effect, age-invariant factors, and interindividual differences based on the cross-sectional design.

## Limitations and Future Directions

Before conclusion, there are some limitations of this study worth noting. First, regarding the current result showing no initial age effect for the follow-up results. One explanation for this phenomenon may be that an average interval of 2 years is not long or sensitive enough to detect actual differential *aging* effects for people with different initial ages. Nevertheless, we still think even with such a brief follow-up interval, this study is still worthy because if there is an obvious age effect based on the cross-sectional evidence, then there could be some abrupt change even over a rather short period of time for older people as compared with younger people. The results of the current study at least suggest that over a short period of time, older people exhibited a similar aging change rate as younger people. However, a future study using a much longer follow-up interval (e.g., over 5 years or more) is warranted to ensure for following how long a period of time, the specific aging trajectory would show an “initial age” effect as evident in the cross-sectional study.

Second, the current study recruited participants with the age range of 20–78 years old, which might overlook the much older people’s data. Nevertheless, we would like to emphasize that this study did not specifically aim to study “very old” people, but across the entire adult lifespan, thus recruiting participants with a more evenly age distribution (20–29 years: *n* = 20; 30–49 years: *n* = 24; 50–64 years: *n* = 36; 65–78 years: *n* = 22) is necessary.

Third, although we originally recruited 274 participants for time point 1, unfortunately, only 107 of them completed the two time points’ examinations, and subsequently, due to MRI technical issues, only 102 participants’ data were included for the analyses. To test if the remaining sample size of 102 could nevertheless yield sufficient statistical power for the observed significant correlation coefficient, we estimated the required minimum sample size based on Lachin’s (1981) method (see also Hulley et al., 2013). Suppose we wish to detect a simple correlation *r* (*r* = 0.283: the critical value for Bonferroni corrected *p* = 0.004 in this study) of *N* observations using a two-sided test, 5% significance level test (α = 0.05) with 80% power (β = 0.2), the required sample size is approximate 96 (*n* = 96). Therefore, the current correlation results based on 102 participants reach above 80% power. Despite this still sufficient power, future study with more and much older people is encouraged.

Fourth, the current study only acquired two time point measurements which limited the power to model longitudinal findings and allowed for more sophisticated analysis such as mixed modeling (Mills and Tamnes, 2014). Future studies with more than two time points are warranted in order to characterize the longitudinal change more analytically.

Fifth, the possibility for explaining no initial age effect for the relationship between changes in brain structures and cognitive control function may be due to practice effect (Salthouse, 2010), which masked the aging trajectories. Especially, the current study used identical versions of the MoCA and cognitive tests at the two time points over a short period of 2 years which could create strong practice effects. In the current results, an increased score of 2 points for MoCA at TP2 seemed to suggest this possibility. However, even though we cannot exclude the practice effect in the current data, at least we can speculate that people of different ages can benefit from practice over a period of an average of 2 years with similar or maybe even larger magnitude thus elevating the aging effect. Nevertheless, future studies should try to minimize the problem of practice effects by adopting different versions of the equivalent tests in order to examine the actual longitudinal age changes.

Finally, in this study, we measured GMV by means of the popularly used FreeSurfer software (see Text Footnote 1, Center for Biomedical Imaging, Charlestown, MA, United States), which is an automated surface-reconstruction scheme. That is, ROIs of the CCN were extracted using neuroanatomical labels in the Desikan–Killiany Atlas to map on a cortical surface model (see the previous “Materials and Methods” section for details). Other methods for measuring GMV is to calculate for every volumetric point within the cortex rather than on the surface (Yezzi and Prince, 2003; Hutton et al., 2008), such as the method of voxel-based morphometric differences in the brain (VBM; Ashburner et al., 2003). Literature has shown that different measurements of brain structures yielded different conclusions regarding normal aging (Salat et al., 2004) and cognitive performance (Dickerson et al., 2008). Therefore, in order to generalize the current findings, we also performed VBM analyses using the SPM8 toolbox^4^. The results of this alternative method showed similar patterns as reported here with FreeSurfer (see Supplementary Tables 3, 4). Therefore, the current finding is not methodology specific. Nevertheless, future studies are encouraged to directly compare different analytical pipelines such as VBM and FSL in order to rule out technical or biased artifacts that could be generated by using a single pipeline.

## Conclusion

To conclude, the current study directly compared cross-sectional and longitudinal data collected from the same sample and found the two sets of analysis results’ patterns on cognitive control function differed significantly, whereas processing speed remained similar. That is, while we observed several significant and trends of associations between cross-sectional GMV and cognitive control function, we observed much fewer significant relationships between longitudinal GMV change with cognitive function change (e.g., right par_OPC associated with 1-back *d*′). This result warrants the importance of longitudinal research for aging studies to elucidate actual *aging* processes in cognitive control function.

## Data Availability Statement

The raw data supporting the conclusions of this article will be made available by the authors, without undue reservation, to any qualified researcher.

## Ethics Statement

The studies involving human participants were reviewed and approved by Research Ethics Committee of the National Cheng Kung University, Tainan, Taiwan (Contract No. 104–004). The patients/participants provided their written informed consent to participate in this study. The patients/participants provided their written informed consent to participate in this study.

## Author Contributions

SH contributed to the grant resources, the design of the experiments, data processing, and preparation of the manuscript. M-HY contributed to the data collection and analysis, and preparation of figures and tables. Both authors contributed to the article and approved the submitted version.

## Conflict of Interest

The authors declare that the research was conducted in the absence of any commercial or financial relationships that could be construed as a potential conflict of interest.

## Footnotes

1 http://surfer.nmr.mgh.harvard.edu/

2 https://surfer.nmr.mgh.harvard.edu/fswiki/CorticalParcellation

3 Please note, in the study by Schippling et al. (2017), they regressed out total intracranial volume (TIV) before estimating the annual brain volume loss. To avoid this possible confound also occurred in our observations, we likewise additionally regressed out TIV for all the correlational analyses. The new results with further controlling for TIV showed similar patterns as reported (please see Supplementary Tables 1, 2 for details).

4 https://www.fil.ion.ucl.ac.uk/spm/software/spm8/
