# Supplementary material for: Two-Year Follow-Up Study of the Relationship Between Brain Structure and Cognitive Control Function Across the Adult Lifespan
Source: Front Aging Neurosci. 2021 Jun 1;13:655050. doi: 10.3389/fnagi.2021.655050 (PMC8205153; doi:10.3389/fnagi.2021.655050)
Supplement: Supplementary file 1 [file Table_1.docx]

Supplementary Table 1. Cross-sectional correlation table (n = 102)

|  |  | cross sectional GMV (TP1)correlation with | | | | |  | cross sectional GMV(TP1)correlation with | | | | | |
| --- | --- | --- | --- | --- | --- | --- | --- | --- | --- | --- | --- | --- | --- |
|  |  | age | behavior(covariate: sex, edu, BDI-II,TIV) | | | |  | behavior(covariate: age, sex, edu, BDI-II,TIV) | | | | |  |
|  |  |  | speed | shifting | inhibition | memory |  | speed | shifting | inhibition | memory |  |  |
| dACC | rosAntCG_L | -0.418* | ns | 0.246(SWI) | -0.247(SSRT) | 0.234(1back) |  | ns | ns | ns | ns |  |  |
|  | rosAntCG_R | -0.298* | -0.257(GPTR)  -0.260(GPTL) | ns | -0.256(SSRT) |  |  | ns | ns | ns | ns |  |  |
| DLPFC | infF-parOPC_L | -0.470* | -0.277(TMTA) -0.251(GPTR)  -0.240(GPTL) | -0.220(TMTB) |  | 0.232(1back) |  | 0.253(goRT) | ns | ns | ns |  |  |
|  | infF-parOPC_R | -0.548* | -0.364(TMTA)*  -0.270(GPTR)  -0.324(GPTL)* | ns | -0.288(SSRT)* | 0.220(1back) |  | ns | ns | ns | ns |  |  |
|  | rosMidF_L | -0.707* | -0.325(TMTA)*  -0.390(GPTR)*  -0.438(GPTL)*  -0.361(goRT)* | ns | -0.237(SSRT) | 0.352(2back)* |  | ns | ns | ns | ns |  |  |
|  | rosMidF_R | -0.647* | -0.250(TMTA)  -0.298(GPTR)*  -0.337(GPTL)*  -0.244(goRT) | 0.272(SWI) | -0.213(SSRT) | 0.252(2back) |  | ns | 0.294(noninfSWI)*  0.202(TMTB) | ns | ns |  |  |
| DPC | infP_L | -0.455* | -0.213(TMTA)  -0.288(GPTR)*  -0.302(GPTL)*  -0.312(goRT)* | ns | ns | ns |  | ns | ns | ns | ns |  |  |
|  | infP_R | -0.548* | -0.372(TMTA)*  -0.474(GPTR)*  -0.464(GPTL)*  -0.218(goRT) | -0.250(TMTB) | ns | 0.235(2back) |  | -0.248(GPTR) | ns | ns | ns |  |  |
|  | supP_L | -0.527* | -0.243(GPTR)  -0.366(GPTL)* | ns | -0.219(SSRT) | ns |  | ns | ns | ns | ns |  |  |
|  | supP_R | -0.523* | -0.220(TMTA)  -0.232(GPTR)  -0.327(GPTL)* | 0.219(SWI)  -0.201(TMTB) | -0.328(SSRT)* | 0.211(2back) |  | ns | ns | ns | ns |  |  |
|  | precuneus_L | -0.610* | -0.252(TMTA)  -0.276(GPTR)  -0.400(GPTL)*  -0.229(goRT) | -0.201(TMTB) | ns | 0.219(2back) |  | ns | ns | ns | ns |  |  |
|  | precuneus_R | -0.524* | -0.310(TMTA)*  -0.357(GPTR)*  -0.445(GPTL)* | -0.289(TMTB)* | ns | ns |  | ns | ns | ns | ns |  |  |

*p < 0.004 (Bonferroni corrected); GMV: gray matter volume; edu: education; L: left; R: right hemisphere; dACC : dorsal anterior cingulate cortex; rosAntCG : rostral anterior cingulate gyrus; DLPFC: dorsolateral prefrontal cortex; infF-parOPC: pars opercularis of the inferior frontal gyrus; rosMidF: rostral middle frontal gyrus; DPC: dorsal parietal cortex; infP: inferior parietal cortex (infP); supP: superior parietal cortex; TMT-A: Trail Making Test – Form A; GPT_L: Grooved Pegboard Test, left hand; GPT_R: Grooved Pegboard Test, right hand; TMT-B: Trail Making Test – Form B; SWI: switch cost in informative cue condition; noninfSWI: switch cost in non-informative cue condition; SSRT: Stop-signal reaction time; 2-back: 2-back task’s sensitivity; 1-back: 1-back task’s sensitivity.
