# Supplementary material for: Two-Year Follow-Up Study of the Relationship Between Brain Structure and Cognitive Control Function Across the Adult Lifespan
Source: Front Aging Neurosci. 2021 Jun 1;13:655050. doi: 10.3389/fnagi.2021.655050 (PMC8205153; doi:10.3389/fnagi.2021.655050)
Supplement: Supplementary file 2 [file Table_2.docx]

Supplementary Table 2. Longitudinal correlation table (n = 102)

|  |  | longitudinal ΔGMV correlation with | | | | |  | longitudinal ΔGMV correlation with | | | | | |
| --- | --- | --- | --- | --- | --- | --- | --- | --- | --- | --- | --- | --- | --- |
|  |  | age (tp1) | behaviorΔ(covariate: sex, edu, BDI-II,TIV) | | | |  | behaviorΔ (covariate: age, sex, edu, BDI-II,TIV) | | | | |  |
|  |  |  | speed | shifting | inhibition | memory |  | speed | shifting | inhibition | memory |  |  |
| dACC | rosAntCG_L | 0.288* | ns | ns | ns | ns |  | ns | ns | ns | ns |  |  |
|  | rosAntCG_R | ns | ns | ns | ns | 0.223(2-back)  -0.200(MIX) |  | ns | ns | ns | 0.224(2-back)  -0.201(MIX) |  |  |
| DLPFC | infF-parOPC_L | ns | ns | ns | 0.210(SSRT) | ns |  | ns | ns | 0.202(SSRT) | ns |  |  |
|  | infF-parOPC_R | ns | ns | ns | 0.220(SSRT) | 0.284(1-back)* |  | ns | ns | 0.231(SSRT) | 0.282(1-back) |  |  |
|  | rosMidF_L | ns | ns | 0.227(noinfsw) | ns | ns |  | ns | 0.221(noninfswi) | ns | ns |  |  |
|  | rosMidF_R | ns | ns | 0.224(noinfswi) | ns | ns |  | ns | 0.205(noninfswi) | ns | ns |  |  |
| DPC | infP_L | ns | ns | ns | ns | ns |  | ns | ns | ns | ns |  |  |
|  | infP_R | ns | ns | ns | ns | ns |  | ns | ns | ns | ns |  |  |
|  | supP_L | ns | ns | ns | ns | 0.268(1-backd') |  | ns | ns | ns | 0.266(1-back) |  |  |
|  | supP_R | ns | ns | ns | ns | ns |  | ns | ns | ns | ns |  |  |
|  | precuneus_L | ns | ns | ns | ns | ns |  | ns | ns | ns | ns |  |  |
|  | precuneus_R | ns | ns | ns | ns | ns |  | ns | ns | ns | ns |  |  |

*p < 0.004 (Bonferroni corrected); GMV: gray matter volume; edu: education; L: left; R: right hemisphere; dACC : dorsal anterior cingulate cortex; rosAntCG : rostral anterior cingulate gyrus; DLPFC: dorsolateral prefrontal cortex; infF-parOPC: pars opercularis of the inferior frontal gyrus; rosMidF: rostral middle frontal gyrus; DPC: dorsal parietal cortex; infP: inferior parietal cortex (infP); supP: superior parietal cortex; TMT-A: Trail Making Test – Form A; GPT_L: Grooved Pegboard Test, left hand; GPT_R: Grooved Pegboard Test, right hand; TMT-B: Trail Making Test – Form B; SWI: switch cost in informative cue condition; noninfSWI: switch cost in non-informative cue condition; SSRT: Stop-signal reaction time; 2-back: 2-back task’s sensitivity; 1-back: 1-back task’s sensitivity.
