# Supplementary material for: Two-Year Follow-Up Study of the Relationship Between Brain Structure and Cognitive Control Function Across the Adult Lifespan
Source: Front Aging Neurosci. 2021 Jun 1;13:655050. doi: 10.3389/fnagi.2021.655050 (PMC8205153; doi:10.3389/fnagi.2021.655050)
Supplement: Supplementary file 3 [file Table_3.docx]

Supplementary Table 3. Cross-sectional correlation table (n = 102) using voxel-based morphometric (VBM) approach

|  |  | cross sectional GMV (TP1)correlation with | | | | |  | cross sectional GMV(TP1)correlation with | | | | | |
| --- | --- | --- | --- | --- | --- | --- | --- | --- | --- | --- | --- | --- | --- |
|  |  | age | behavior(covariate: sex, edu, BDI-II) | | | |  | behavior(covariate: age, sex, edu, BDI-II) | | | | |  |
|  |  |  | speed | shifting | inhibition | memory |  | speed | shifting | inhibition | memory |  |  |
| dACC | rosAntCG_L | -0.779* | -0.513(GPTL)*  -0.429(GPTR)*  -0.449(TMTA)*  -0.366(goRT)* | -0.287(TMTB)*  0.209(SWI) | -0.350(SSRT)* | 0.298(2back)* |  | ns | ns | ns | ns |  |  |
|  | rosAntCG_R | -0.773* | -0.509(GPTL)*  -0.426(GPTR)*  -0.445(TMTA)*  -0.362(goRT)* | -0.283(TMTB)*  0.204(SWI) | -0.345(SSRT)* | 0.302(2back)* |  | ns | ns | ns | ns |  |  |
| DLPFC | infF-parOPC_L | -0.777* | -0.513(GPTL)*  -0.443(GPTR)*  -0.464(TMTA)*  -0.387(goRT)* | -0.290(TMTB)* | -0.374(SSRT)* | 0.294(2back)* |  | ns | ns | ns | ns |  |  |
|  | infF-parOPC_R | -0.750* | -0.504(GPTL)*  -0.428(GPTR)*  -0.440(TMTA)*  -0.376(goRT)* | -0.281(TMTB) | -0.361(SSRT)* | 0.287(2back)* |  | ns | ns | ns | ns |  |  |
|  | rosMidF_L | -0.794* | -0.510(GPTL)*  -0.444(GPTR)*  -0.460(TMTA)*  -0.378(goRT)* | 0.206(SWI)  -0.291(TMTB)* | -0.352(SSRT)* | 0.295(2back)* |  | ns | ns | ns | ns |  |  |
|  | rosMidF_R | -0.770* | -0.508(GPTL)*  -0.434(GPTR)*  -0.435(TMTA)*  -0.361(goRT)* | 0.199(SWI)  -0.275(TMTB) | -0.351(SSRT)* | 0.297(2back)* |  | ns | ns | ns | ns |  |  |
| DPC | infP_L | -0.733* | -0.501(GPTL)*  -0.440(GPTR)*  -0.485(TMTA)*  -0.451(goRT)* | -0.349(TMTB)* | -0.423(SSRT)* | 0.314(2back)* |  | -0.268(goRT) | ns | -0.245(SSRT) | ns |  |  |
|  | infP_R | -0.714* | -0.503(GPTL)*  -0.439(GPTR)*  -0.456(TMTA)*  -0.437(goRT)* | -0.329(TMTB)* | -0.414(SSRT)* | 0.304(2back)* |  | -0.249(goRT) | ns | -0.235(SSRT) | ns |  |  |
|  | supP_L | -0.735* | -0.492(GPTL)*  -0.432(GPTR)*  -0.478(TMTA)*  -0.437(goRT)* | -0.333(TMTB)* | -0.421(SSRT)* | 0.310(2back)* |  | -0.245(goRT) | ns | -0.242(SSRT) | ns |  |  |
|  | supP_R | -0.716* | -0.491(GPTL)*  -0.428(GPTR)*  -0.459(TMTA)*  -0.435(goRT)* | -0.325(TMTB)* | -0.415(SSRT)* | 0.305(2back)* |  | -0.246(goRT) | ns | -0.236(SSRT) | ns |  |  |
|  | precuneus_L | -0.725* | -0.505(GPTL)*  -0.435(GPTR)*  -0.471(TMTA)*  -0.458(goRT)* | -0.329(TMTB)* | -0.416(SSRT)* | 0.305(2back)* |  | -0.280(goRT) | ns | -0.237(SSRT) | ns |  |  |
|  | precuneus_R | -0.716* | -0.502(GPTL)*  -0.434(GPTR)*  -0.458(TMTA)*  -0.453(goRT)* | -0.325(TMTB)* | -0.409(SSRT)* | 0.303(2back)* |  | 0.274(goRT) | ns | -0.227(SSRT) | ns |  |  |

*p < 0.004 (Bonferroni corrected); GMV: gray matter volume; edu: education; L: left; R: right hemisphere; dACC : dorsal anterior cingulate cortex; rosAntCG : rostral anterior cingulate gyrus; DLPFC: dorsolateral prefrontal cortex; infF-parOPC: pars opercularis of the inferior frontal gyrus; rosMidF: rostral middle frontal gyrus; DPC: dorsal parietal cortex; infP: inferior parietal cortex (infP); supP: superior parietal cortex; TMT-A: Trail Making Test – Form A; GPT_L: Grooved Pegboard Test, left hand; GPT_R: Grooved Pegboard Test, right hand; TMT-B: Trail Making Test – Form B; SWI: switch cost in informative cue condition; noninfSWI: switch cost in non-informative cue condition; SSRT: Stop-signal reaction time; 2-back: 2-back task’s sensitivity; 1-back: 1-back task’s sensitivity
